# Scanning Probe Microscopy Facility for Operando Study of Redox Processes on Lithium ion Battery Electrodes

**DOI:** 10.3389/fchem.2021.505876

**Published:** 2021-04-15

**Authors:** W. J. Legerstee, M. Boekel, S. Boonstra, E. M. Kelder

**Affiliations:** ^1^ Storage of Electrochemical Energy, Radiation Science and Technology, Applied Sciences, Delft University of Technology, Delft, Netherlands; ^2^ Automotive Engineering, Engineering and Applied Sciences, Rotterdam University of Applied Sciences, Rotterdam, Netherlands

**Keywords:** scanning probe microscopy, lithium ion battery, operando, atomic force microscope, silicon lithium alloying, batteries

## Abstract

An Atomic Force Microscope (AFM) is combined with a special designed glovebox system and coupled to a Galvanostat/Potentiostat to allow measurements on electrochemical properties for battery research. An open cell design with electrical contacts makes it possible to reach the electrode surface with the cantilever so as to perform measurements during battery operation. A combined AFM-Scanning Electro-Chemical Microscopy (AFM-SECM) approach makes it possible to simultaneously obtain topological information and electrochemical activity. Several methods have been explored to provide the probe tip with an amount of lithium so that it can be used as an active element in a measurement. The “wet methods” that use liquid electrolyte appear to have significant drawbacks compared to dry methods, in which no electrolyte is used. Two dry methods were found to be best applicable, with one method applying metallic lithium to the tip and the second method forming an alloy with the silicon of the tip. The amount of lithium applied to the tip was measured by determining the shift of the resonance frequency which makes it possible to follow the lithiation process. A FEM-based probe model has been used to simulate this shift due to mass change. The AFM-Galvanostat/Potentiostat set-up is used to perform electrochemical measurements. Initial measurements with lithiated probes show that we are able to follow ion currents between tip and sample and perform an electrochemical impedance analysis in absence of an interfering Redox-probe. The active probe method developed in this way can be extended to techniques in which AFM measurements can be combined with mapping electrochemical processes with a spatial resolution.

## Introduction

The automotive industry with its high demands has an enormous driving force behind the large growth of the battery market. Due to practical requirements, there is a need for faster charging solutions and a large difference in charging strategies is offered to charge vehicles. Fast charging becomes more common and contact-less charging is around the horizon. Large variations in charging conditions lead to degradation of the battery and faster aging (Nagpure et al., 2013). At the same time, new battery materials with a higher gravimetric and volumetric energy density are being sought, and these requirements must also be combined with automotive requirements such as high stability and long battery life.

On the one hand, improvements to the current lithium ion (Li-ion) cells are being sought, but more and more research is being done into possibilities beyond Li-ion batteries, for example sodium-ion and magnesium-ion batteries. This increases the need for specialized research into rechargeable batteries and the need for new and powerful characterization methods.

Several research groups have developed scanning probe microscopy techniques to investigate electrochemical processes (Arreaga-Salas et al., 2012; Kim et al., 2018; Benning et al., 2019; Zhang et al., 2020), and a commercial AFM-SECM instrument has recently become available which is suitable for battery research (Bruker, 2020). However, all these scanning probe techniques are used to *analyze* battery processes on micro to nano level, while charge and discharge conditions are *controlled* at bulk level. Therefore, in this study we investigated the possibility of using the probe as an active component during the measurement, and so, makes it possible to tune charge and discharge parameters on the local scale. A set-up has been developed for this purpose in which an AFM has access to an open battery cell under controlled conditions. Several techniques have been investigated to provide the probe with lithium so that it can operate as counter electrode and participate as part of the dynamic interphase processes on a controlled way.

Significant battery aging processes take place at the interfaces between the electrode and the electrolyte. A Solid Electrolyte Interphase (SEI) forms at this interface, which is related to processes responsible for aging and loss of capacity. The capacity loss consists of a reversible and an irreversible part, both contributing to the impedance of the interphase (Lam et al., 2011). With bulk-level Electrochemical Impedance Spectroscopy (EIS) it is possible to locate these processes and distinguish between the contribution of reversible and irreversible processes, but the relationship between the irreversible processes and local electrode surface properties, such as morphology, local composition, grain size and shape, crystal orientations, etc. is poorly known. Hence, combining AFM and non-faradaic EIS provides a powerful method to investigate local processes at the nanoscale and get more insight into transport physics as a function of spatial coordinates.

## Scanning Probe Microscopy

Scanning probe microscopy (SPM) is a collective term for high resolution measurements in which the surface of an object is scanned with a very small probe to produce a three-dimensional image. After the development of the Scanning Tunneling Microscope (STM) (Binnig and Rohrer, 1983) and the Atomic Force Microscope (AFM) (Binnig et al., 1986), many variations on this highly sensitive measuring technique have arisen, among which Scanning Capacitance Microscopy (SCM), Conductive AFM (C-AFM) and Kelvin Probe Force Microscopy (KPFM). The basis of the many variants is AFM, where attractive and repulsive forces between a very sharp tip and the sample surface are used to study surface morphologies. In general, for all these AFM-derivative measurement methods, the distance between the probe and the sample is determined by measuring small changes that occur in a controlled process parameter between surface and probe. These changes are converted into corrective movements of the probe that ensure that the probe accurately follows the surface. This technique can be used to create a 3D surface scan of all conceivable and measurable parameters between sample and probe with resolutions up to the nanometer scale (Nagpure et al., 2010; Amanieu et al., 2015; Kim et al., 2018; Benning et al., 2019; Mahankali et al., 2019).

In principle, the AFM-probe can be used for complex electrochemical measurements, such as impedance measurements on a very local scale. O'Hayre et al. (2004a) performed quantitative impedance measurements in which, by pressing the tip firmly on the sample, a local impedance scan in contact mode can be performed. They developed a model that describes the impedance of the probe surface and takes into account the resistance of the tip, the interfacial resistance (resistance to the faradaic charge transfer), the double layer capacitance and the distribution of the resistance from the probe tip into the sample (O'Hayre et al. 2004b). When the force is brought above a certain (sample-dependent) threshold value and it is kept as constant as possible over all measuring points, it is possible to perform and map SPM impedance measurements to investigate frequency-dependent phenomena on the nanometer scale.

A well-known SPM technique where electrochemical processes between tip and sample are mapped is Scanning Electrochemical Microscopy (SECM) (Nanosensors, 2020; Figure 3; Polcari et al., 2016; Bard et al., 1989). A glass pipet-like probe that consists of a metal core, the Ultra-Micro-Electrode (UME), is immersed in a solution, scans the sample surface and measures the current that results from redox reactions that takes place. The current can be measured as a function of the probe X-Y position which creates a two dimensional image map (Wittstock et al., 2007). Alternating Current Scanning Electrochemical Microscopy (ac-SECM) is a form of SECM in which a sinusoidal bias voltage is applied to the UME to indirectly measure the impedance response of the sample under investigation for a single ac frequency (Diakowski and Ding, 2007; Eckhard et al., 2007). To obtain impedance information over a wider frequency range, a combination of SECM and EIS called “Scanning Electrochemical Impedance Microscopy” (SEIM) has proven to be a powerful technique for investigating local electrochemical changes (Bandarenka et al., 2013). However, with all these methods, amperometric measurements are made between the UME and the sample in the presence of redox mediators and/or redox probes. Recently, from the bio-electrochemistry field, Valiūnienė et al. (2019), avoided the use of redox mediators and probes and demonstrated a redox probe-free approach to SEIM on conductive and non-conductive surfaces. To reduce the duration of the measurements, the SEIM technique was hybridized with Fast-Fourier Transform based EIS (FFT-EIS) which makes it possible to simultaneously measure up to 50 single sine-signals. The resulting non-faradaic FFT-SEIM application is used in the field of bio-electrochemistry for *in-situ* and operando evaluation of electrochemical processes (Morkvenaite-Vilkonciene et al., 2017; Valiūnienė et al., 2020).

### SPM for Battery Research

Many research groups have focused on investigating battery properties using AFM. In the simplest method, the battery is dismantled in a protected glove box and samples are rinsed clean, after which they can be analyzed by AFM (Nagpure et al., 2010; Demirocak and Bhushan 2015; Yang et al., 2017; Kim et al., 2018; Sakai et al., 2019). A major drawback of this method is that the condition of the samples can be affected, which may result in loss of important properties. This type of research is therefore limited to mechanical and morphological techniques, such as analyzing changes in surface morphology, elasticity and hardness through battery use, and measuring changes in the material structure.

More and more researchers are using *in-situ* measurement methods (Doi et al., 2008; Inaba et al., 2011; Demirocak and Bhushan 2014; Wang et al., 2014; Huang et al., 2018; Benning et al., 2019; Mahankali et al., 2019) where information about interface processes can be investigated without influencing them. In general, to perform *in situ* measurements, the battery process is temporarily or completely stopped, after which measurements are taken. This method is useful, inter alia, for investigating the origin and development of the SEI on the electrodes.

The most advanced and complex way of measuring is achieved by operating the battery during the measurement, the so-called *operando* measurements (Breitung et al., 2016; Wang et al., 2017; Zhang et al., 2020). By performing a measurement this way, the dynamic processes that occur can be followed real-time and related to all kinds of battery parameters and settings, bringing this type of measurements closest to the practical behavior of the battery. The facility and techniques discussed in this article have been developed to allow *operando* measurements on batteries, taking it one step further by also including the probe as part of the measurement.

### AFM tip as an Active Element

By creating battery operation between the probe tip and the sample in a controlled manner, a powerful analysis method is added to the range of possibilities that AFM offers. This technique opens the way to simultaneous monitoring of morphological, mechanical, electrical, and electrochemical properties and processes. To achieve this, it is necessary to provide the tip with a small amount of lithium. In fact the tip will function as an active part of a battery.

**FIGURE 1:**
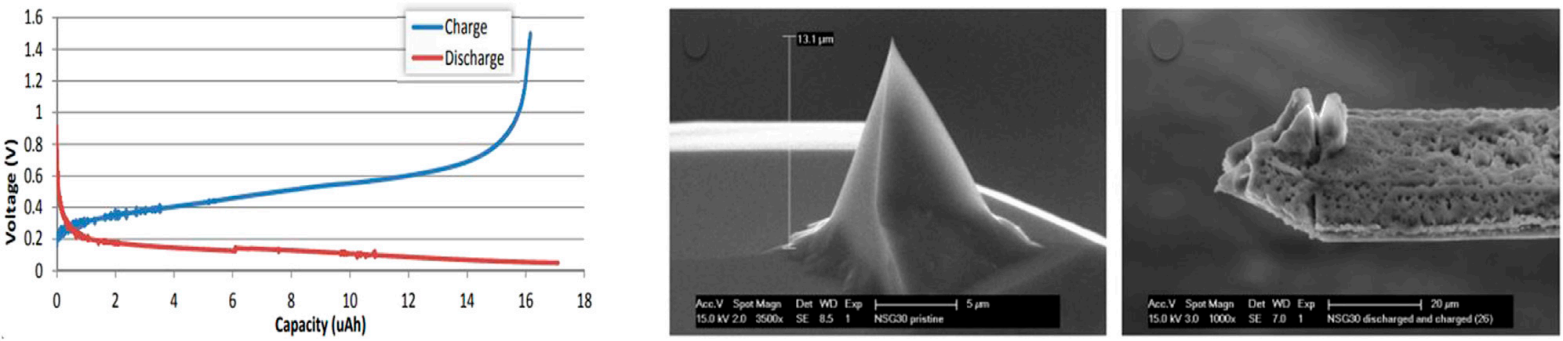
Left: Discharge (red) and charge (blue) curve of silicon probe vs. metallic lithium. Middle: pristine tip. Right: Probe after discharge and charge procedure.

During a preliminary investigation in our laboratory we looked at the possibility of bringing lithium into the probe by alloying a Silicon tip with Lithium (Garcia-Tamayo, 2014). The principle has been demonstrated but many practical problems were encountered. Measurements were performed without the use of a controlled glovebox environment. A liquid measuring cell has been used that was covered with a membrane and flushed with Argon gas, and which offers protection against contamination for a short time (ND-MDT liquid cell). An uninsulated AFM probe (ND-MDT NSG03) was used and measurements were made with the entire chip immersed in electrolyte (1M LiPF_6_ in EC/DMC 1:1), electro-chemically prepared as half-cell vs. metallic lithium. Figure 1 (left) shows a charge-discharge curve that was measured by the use of a Potentiostat/Galvanostat (Metrohm Autolab PG-STAT302F). As a result, lithiation took place over the entire chip, making the measurement not properly controlled and even lithiation resulted in distorted tips due to silicon expansion, see Figure 1 (right).

The conclusion of this exploratory study was that in principle it is possible to store lithium in the tip and these tips can also be stored over a longer period of time without degrading (Garcia-Tamayo, 2014). In fact, although the results were not conclusive, this preliminary study showed that it is possible to alloy lithium with the silicon of the AFM chip (including the cantilever and tip), and electrochemically take it out again. The aim of our research is therefore to make the process controllable and manageable and to investigate how a lithiated probe can contribute to operando measurements of battery materials.

## Experimental set up

For carrying out battery research with an AFM, setups have been realized by various research groups and even an advanced commercial device is available (Bruker, 2020). Here we describe a self-assembled and tailor-made setup that is affordable compared to the commercial offer and also allows a lot of flexibility and adaptability.

### Glovebox Environment

Battery materials are very sensitive to contamination with water and oxygen and therefore an AFM measurement must be performed under protected conditions. Various methods are possible to achieve this. At first a gas-tight and flushable sample holder can be integrated with the probe holder of the AFM equipment. A disadvantage may be that the sample room is very complex, but this keeps the AFM measuring equipment easily accessible and usable for other research that does not require a protected atmosphere. A simple alternative is to use so-called glove bags with which the AFM equipment can be enclosed and placed under Argon atmosphere. Because the relatively poor level of protection against contamination, this mode of operation is inconvenient for long term measurements on batteries. The highest protection can be obtained by placing the AFM equipment in a glovebox. The disadvantage is that all necessary connections for control and measurement have to be entered into the glovebox and that the AFM is only accessible through the gloves of the glovebox. The advantage is the great flexibility in designing the sample holder and the use of the wide range of probes that are commercially available.

It is desirable to set up an AFM as vibration-free as possible. Preventing vibrations increases the accuracy with which measurements can be taken. In order to be able to perform measurements in the desired resolution of 10–200 nm, vibrations must be prevented as much as possible and any vibration damping measures must be taken.

A special glovebox has been designed which includes all requirements for AFM measurements and makes it possible to probe inside open exposed batteries. The designed facility for AFM on batteries consists of a cube-shaped glovebox which is mounted gastight on a heavy stone table, so that the vibration-sensitive AFM stands directly on a stable surface. The connections required for purifying and circulating the inert argon atmosphere through the glovebox are equipped with bellows couplings, preventing the transmission of vibrations from the pump and blower of the purifier unit. To eliminate almost all remaining vibrations, the AFM set-up is placed on an active vibration isolation system that absorbs vibrations in the range of 0.2–200 Hz (Accurion Halcyonics_i4). During sensitive measurements, even the gas flow through the box can cause vibrations, and therefore a bypass system is placed between the gas inlet and outlet. This allows the gas to flow partly through the box and partly through the bypass so that the flow along the AFM can be adjusted such that no disturbances of the measurements occur. During measurements, the gas flow can be reduced while the purity of the atmosphere stays at the desired level for at least 72 h (<1 ppm moisture and oxygen). Test measurements with calibration samples have shown that a scanning resolution of at least 10 nm is achievable under these conditions.

### AFM Coupled With Galvanostat/Potentiostat

The AFM equipment selected for this project is the NT-NDT NTegra P9, a very open instrumental design where access to the sample space can be obtained without obstacles. The AFM equipment consists of two parts, the controller unit, which is located outside the glovebox, and the measuring device, which is located inside the glovebox. All necessary connections for the AFM measuring device have been brought into the glovebox via special connector feed-throughs. The required light for the optical microscope is coupled inwards via a fiber optic connection and a vacuum tight USB port is used for bringing the signals of a digital camera to the computer which allows live video of a scanning cantilever during the measurements.

The optical microscope (Opta 2.0x mini) can be turned away for making excess to the probe and sample holder. The AFM is equipped with a three-legged scanning head which can easily be placed and removed. The more limited conveniences caused by working with gloves are thus minimized while still keeping very accurate measurements possible. This AFM equipment is provided with an internally bias voltage source and a highly accurate current meter, the so-called *iprobe*. The internal circuit can be adjusted via the user interface of the NTegra software (NOVA px), whereby both the tip and the sample can be connected to the internal voltage source, the internal ground, but also to an external connector. To generate more measurement options and enable advanced battery analysis, the connections of a galvanostat/potentiostat (Metrohm Autolab PG-STAT302F) have been brought into the glovebox via coaxial cables, enabling impedance analysis, cyclic voltammetry, charge and discharge procedures and many other electrochemical measurements.

### AFM Accessible Cell

**FIGURE 2:**
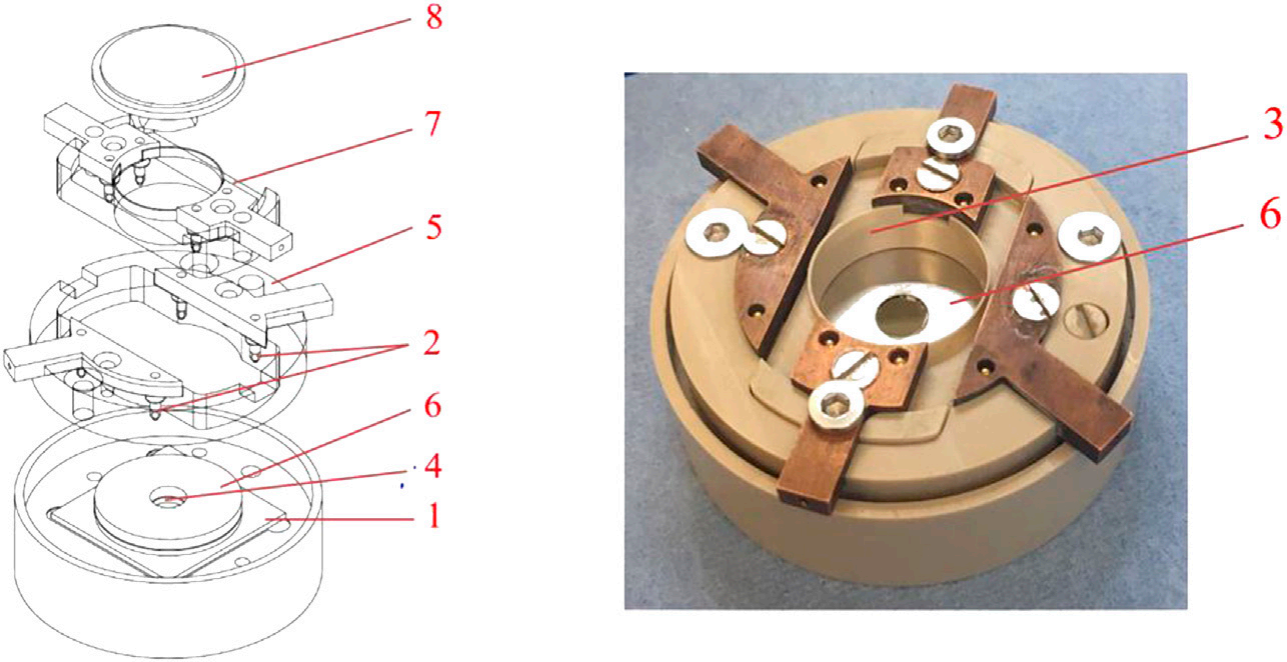
The Atomic Force Microscope accessable battery cell (AFMAB). 1) Sample substrate; 2) spring pin connections; 3) Liquid tray; 4) Separator; 5) Working electrode connector; 6) Lithium disc with hole (larger here then in case of real measurements); 7) Counter electrode connector; 8) Nose piece (probe holder).

In order to be able to perform AFM measurements on the electrode materials within a working battery, a special open-cell sample holder has been developed. Figure 2 shows a cross section and a “battery prepared” photo of the *“Atomic Force Microscope Accessible Battery cell”* (AFMAB). As can be seen in the expanded image, the AFMAB consists of a liquid-tight housing on which all parts of a battery can be stacked. First, a sample substrate (1) is placed in the holder. Electrical contact is obtained with the gold-plated spring pins (2). Separator material is placed on top of the substrate with a small hole (4) in the middle at the position of the sample. Use of a reference electrode is possible by placing it between two stacked separators and leading the electrical contact out with an insulated Kapton® wire. The working part (5) is then screwed onto the housing so that the golden pins (2) make electrical contact with the substrate. On top of the stacked separator a thin lithium anode (6) is placed, again with a small hole (4) at the position of the sample. At last, the counterpart (7) with golden spring pins to the lithium anode is placed on top and connected with screws to tighten the stack of materials. Once the cell has been assembled, the AFMAB can be placed at the measuring position of the AFM and connected to the galvanostat/potentiostat equipment. By applying a desired amount of electrolyte in the liquid-tray (3), a cell is created in which the sample can be used as the working electrode of a battery and can be reached with the AFM. Bulk charge and discharge procedures can be performed until a desired state of the battery is reached, after which *in-situ* measurements can be performed when the processes are paused, or *operando* measurements can be carried out during operation. During the bulk measurements, the cell can be closed with a lid to prevent the electrolyte composition from undergoing major changes due to slow evaporation of the solvents. The cantilever chip is mounted on the crystal nosepiece (8), which is suitable for measurements in liquids, and can be fitted with a rubber membrane that can be used to close the AFMAB cell during measurement to minimize evaporation of the electrolyte.

**FIGURE 3:**
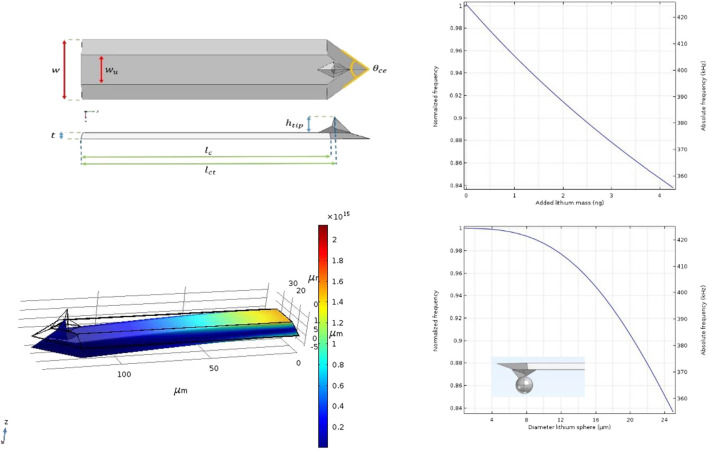
Left: Dimensions and FEM analyses of the CDT-NCHR Diamond coated probe Right: Simulation results of frequency shift as a function of added mass and frequency shift as a function of diameter of a lithium sphere situated at the end of the tip.

## Monitoring the tip Lithiation Process by Shift of Resonance Frequency

In order to make the lithiation process of the tip controllable, a way of monitoring this process was sought. It has been investigated whether resonance frequency shifting of the cantilever in oscillation mode can be used to determine the amount of lithium added to the tip. A model has been set up that describes the relationship between mass change and the shift of the first mode natural resonance frequency, and a simulation of the cantilever oscillation as a function of added mass has been made.

### Theoretical Mechanics of Oscillating Cantilever

Due to the classical mechanics of oscillators a beam clamped on one side behaves like a mass spring system (Poggi et al., 2005; Espinoza Betltran et al., 2006). The complicated beam dynamics of a cantilever spring with small deflections is described by the Euler-Bernoulli equation (Bauchau and Craig, 2009; Payam and Fathipour 2009) and its solutions are given in vibrational modi with their 'eigenfrequencies'. The beam-dynamics for an individual resonance mode can be simplified with the dynamics of a common harmonic oscillator (Boisen et al., 2011), which is given by:

$$f_{0}=\frac{1}{2\pi }\sqrt{\frac{k_{eff}}{m_{eff}}}$$ (1)

For a homogeneously distributed cantilever with no anisotropy of the cantilever material in the beam direction, the effective spring constant *k*_*eff*_ for the first and most important vibrational mode is given by:

$$k_{eff}=\frac{E\cdot h^{3}\cdot w}{4L^{3}}$$ (2)

where *E* is Young’s modulus and *h*, *w*, and *L* are height, width and length of the cantilever, respectively. The effective mass *m*_*eff*_ for the first mode of a homogeneously distributed cantilever is given by:

$$m_{eff}=0,2427\;m_{cantilever}$$ (3)

For a freely oscillating cantilever the resonance frequency *f*_*0*_ changes when a small amount of lithium with mass *m*_*Li*_ is added to the very end of the beam. When we assume that m_eff_ >> m_Li_ and the mass is added at the end of the cantilever, the shifted resonance frequency for the first vibrational mode becomes:

$$f_{Li}=\frac{1}{2\pi }\sqrt{\frac{k_{eff}}{m_{eff}+m_{Li}}}$$ (4)

When we introduce the relative resonance shift *f*_*n*_ as.

$$f_{n}=\frac{f_{Li}}{f_{0}}$$ (5)

It follows from Eqs. 1, 4 that the added amount of lithium depends on *f*_*n*_ and *m*_*eff*_ as:

$$m_{Li}=\frac{f_{n}^{2}}{1-f_{n}^{2}}m_{eff}$$ (6)

With this equation a general relationship has been formulated between the relative resonance shift and the amount of added mass to the cantilever tip.

AFM probes are commercially available in many different shapes and sizes, made from different materials and often provided with a special coating, for instance a reflective or conductive layer. When a thin film of a different material is added to the surface of a cantilever, this will affect the mechanical bending properties and so the vibrational properties of the beam. Depending on the thickness of the layer and the orientation of the anisotropic substrate (Silicon cantilever), additional mechanical stress will occur, which in principle can be determined with an extended version of Stoney's equation (Injeti and Annabattula, 2016). Both this stress and the addition of a layer with its own Young's modulus will influence the bending properties and thus the spring constant of the cantilever.

### Responsivity and Sensitivity

In principle we want to use the AFM probe as a very sensitive mass detection instrument, i.e., a beam-based mass sensor, a technique which is often used for detection and mass determination of molecules in the field of bio-science (Boisen et al., 2011). In our case, a small amount of lithium is added to the very end of the beam during the lithiation process, which changes the effective mass *m*_*eff*_ of the cantilever, resulting in a shift of the first mode resonance frequency. The minimum detectable mass, the *responsivity R*_*c*_ is determined by the ratio between the mass of the cantilever and the free resonance frequency (Zhang and Hoshino 2018):

$$R_{c}=\;\frac{\Delta f_{0}}{\Delta m_{eff}}$$ (7)

When we assume that the change of mass is very small in relation to the mass of the cantilever, the responsivity can be written as derivative of Eq. 1 with respect to the mass of the beam:

$$R_{c}\approx \frac{\partial f}{\partial m_{eff}}=-\frac{f_{0}}{2m_{eff}}\;\;$$ (8)

Eq. 8 clearly indicates that the responsivity can be improved by increasing the resonance frequency or reducing the mass of the cantilever, both attainable by lowering the beam dimensions or reducing the material density. The dimensions of an AFM probe are in the order of micrometers and the probe is often made of Silicon, the sensitivity of this mass sensor is therefore roughly calculated between *10–30 kHz/ng*.

The responsivity can be regarded as a property of the used cantilever in a particular situation which makes it an important value in determining the capability of a probe for use as a mass detection sensor. The same ratio as described by Eq. 7 can be determined when including the instrument properties, such as noise, external vibrations and other influences. This will give us the *sensitivity S*_*inst*_:

$$S_{inst}=\;\frac{\Delta f_{\min}}{\Delta m_{\min}}$$ (9)

which is defined as the minimum input level required to produce an output that overcomes the threshold.

### Influence of Damping

When describing the behavior of a cantilever as a harmonic oscillator, the effect of energy loss has not been taken into account so far. Damping, or the dissipation of kinetic energy from a vibrating cantilever is caused by both intrinsic processes (determined by material properties) and extrinsic processes (determined by the environment). The latter especially requires attention when operating the AFM under ambient conditions because the dominant source of dissipation of kinetic energy is the surrounding medium. For our application, in general, if the damping of the system increases, the resonance frequency decreases, causing the *responsivity R*_*c*_ to decrease. In addition, the resonance peak is broadened (Q-factor decreases), which increases the noise level and so decreases the *sensitivity S*_*ins*_. This makes it challenging to determine the added lithium mass on a cantilever in fluid environments using frequency shifting (Sader, 1998; Bhiladvala and Wang, 2004; Korayem et al., 2012; Payam and Fathipour, 2014).

### Modeling Frequency Shift as a Function of Added Lithium on the tip

Due to the complexity that arises when all parameters and properties of an oscilaiting cantilever are included, the dynamic behavior of a cantilever with added lithium has been simulated with Finite Element Method analysis (FEM) using *Comsol multiphysics*® software package.

As a result of the production process, even the same type of cantilevers show variations in dimensions and shape (Poggi et al., 2005). These variations have an effect on the harmonic vibration properties of the beam, so in fact, no cantilever has the same resonance frequency. Therefore, in order to simulate the harmonic oscillation of a given cantilever, the exact measures must be known. Dimensions, shape and position of the tip are required to calculate the resonance frequency of a particular cantilever. For the example of a FEM analysis given here we used a diamond coated conductive probe (Nanosensors GmbH CDT-NCHR, Nominal resonance frequency 400 kHz, Nominal force constant 80 N/m). The exact dimensions have been determined by measuring the sizes of the tip and cantilever with the use of a scanning electron microscope (SEM: JEOL JSM-IT100). The values stated by the manufacturer have been used for all mechanical material properties needed for the simulation (Silicon, density 2,329 kgm^−3^, E = 170 ✕ 10^9^ Pa, Poison ratio = 0.28; Diamond, density 3,514.9 kgm^−3^, E = 1.1448 ✕ 10^12^ Pa, Poison ratio = 0.068992). The thin layer of diamond that covers the probe has a thickness of 100 nm and the cantilever is provided with an aluminum reflection layer (thickness 30 nm) on top.

**FIGURE 4:**
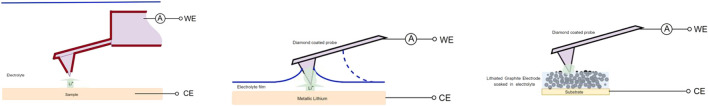
Wet probe lithiation methods. Left: Immersing method; Middle: thin film method; Right: soaked electrode method (reference electrode was connected to CE in all situations).

Figure 4A shows a 3D presentation of the model used for the FEM analysis. The plane at position 0 is actually the plane where the cantilever is attached to the chip and is therefore defined as a fixed plane. A vibrational analysis can be performed on the model whereby the first harmonic resonance frequency is calculated. Figures 4C,D shows the result of the vibration analysis on the cantilever model. The shift of the normalized frequency as a function of the added lithium mass on the end of the tip as well as the frequency shift as a function of the diameter of a (theoretical) lithium sphere are presented. By using Eq. 7, the *responsivity* of the CDT-NCHR can be determined on *R*_*c*_ *= 17,5 kHz/ng*.

## Probe tip Lithiation Methods

The methods that have been explored to supply the tip of the AFM probe with lithium can be divided into methods using liquid electrolyte (further mentioned as wet methods) and methods where no electrolyte is used (dry methods). Table 1 provides an overview of all lithiation methods that have been studied. The main methods are further explained below.

**TABLE 1:** Probe tip lithiation methods.

Method		Way of Lithium transfer	Used probe	Li mass determination by $f_{r}$ shift	efficacy
Wet	Electro chemical plating	Immerse chip in electrolyte	Isolated with exposed end NN-EENP-FM60	Poor responsivity	Reactions with electrolyte disrupt AFM operation
		Immerse tip in electrolyte film	diamond coated CDT-NCHR	Extra cleaning step needed	possibility that cantilever is covered with electrolyte during lithiation
		Immerse tip in electrolyte soaked graphite electrode	diamond coated CDT-NCHR	Extra cleaning step needed	Lithium forms dendrite structure
	Alloying (Li_x_Si)	Immerse tip in electrolyte film	Doped Si NSG30	Extra cleaning step needed	Successful lithiation
		Immerse tip in electrolyte soaked graphite electrode	Doped Si NSG30	Extra cleaning step needed	Successful lithiation
Dry	Mechanical	Adhesion	Platinum coated PtSiNCH	Good responsivity	Successful lithiation
	Electro chemical plating	Contact	diamond coated CDT-NCHR	Good responsivity	Successful lithiation
	Alloying (Li_x_Si)	Contact	Doped Si NSG30	Good responsivity	Successful lithiation
		Contact	Doped Si NSG03	Poor responsivity	Successful lithiation

### Wet Lithiation

In the wet lithiation experiments it was investigated how the tip of the probe can be supplied with lithium by bringing it into contact with electrolyte. Two principles have been explored to provide the tip with lithium, namely the plating method, based on the electrochemical deposition of a layer of lithium on the surface of the tip, and the alloy method, in which the lithium is alloyed with the silicon of the tip.

#### Lithiation of the tip by Immersing the Entire Probe

The possibility of lithering the tip by immersing the entire chip in the electrolyte has been investigated, requiring only the tip to be covered. Therefore, as shown in Figure 4 (left), a conductive and completely insulated probe is used, with only the end of the tip exposed. The choice between insulated probes is very limited, but a probe has been found with a platinum coated tip (NaugaNeedles^TM^ NN-EENP-FM60 Nominal resonance frequency 60 kHz, Nominal force constant 3 N/m), which allows the plating method. The insulation on the cantilever and the carrier-chip makes it possible to completely or partially submerge the chip in electrolyte (LiPF6 EC/DMC 1:1, Sigma Aldrich). An electro-chemical half-cell was created between the AFM probe (working electrode) and a disc of Lithium (counter electrode). By adding a potential an ion current starts to flow through, hence, forcing lithium ions to move to the open tip end and as a result, depositing the exposed end of the tip with lithium.

Basically this method works and lithium is deposited on the tip, but a number of adverse effects occur, making this method practically not usable. Almost immediately after immersing the chip in electrolyte reactions takes place between the electrolyte and the material of the nosepiece and other parts of the equipment. The probe chip, including the reflective gold plating on the cantilever, is also affected, causing the laser reflection signal to become increasingly weaker and completely disappear over time, ultimately rendering probe control impossible. In addition, there is another important reason to preferably measure with a non-submerged cantilever. The large vibration damping effect of liquid on the cantilever will shift the resonance frequency of the cantilever to very low values, which has the consequence that the responsivity to mass change is much lower (see *Influence of Damping*). Determining the added mass of lithium at the tip by measuring the resonance shift is therefore not possible with the desired accuracy.

The method in which the entire tip is immersed, thus appears therefore to be very difficult to implement. In general, we can conclude that the immersion method influences the AFM’s most important measurement principles over time and is not suitable for our purpose. Because of these drawbacks, methods have been investigated in which the cantilever can be kept out of the liquid. If the liquid only covers the tip and does not pass over the cantilever, this offers additional possibilities. This means for our purpose that non-insulated conductive probes can be used, which are widely available commercially and can be selected from a wide variety of material compositions and properties.

#### Lithiation by Immersing the tip in Electrolyte Film

Figure 4 (middle) schematically illustrates a method of lithiating only the tip of the probe by pressing it in a thin deposited electrolyte film in a controlled manner. This method is performed with two types of electrolyte, namely the widely used LiPF6 EC/DMC 1:1 (Sigma Aldrich), and moreover a salt in a liquid state at room temperature, so-called *“Ionic Liquid” (IL) (C*_*8*_ *H*_*11*_ *F*_*6*_ *N*_*3*_ *O*_*4*_ *S*_*2*_ */LiC*_*2*_ *F*_*6*_ *NO*_*4*_ *S*_*2*_ *100:1, Solvionic)*. The use of a diamond coated conductive probe (Nanosensors GmbH CDT-NCHR) (Yu et al., 2018) allows the electrochemical plating method, while the use of a conductive silicon probe (NT-MDT NSG30) is suitable to form a silicon-lithium alloy tip. A disc of metallic lithium (99.99 pure, Sigma Aldrich) was used as a substrate and counter electrode for the lithiation process.

**FIGURE 5:**
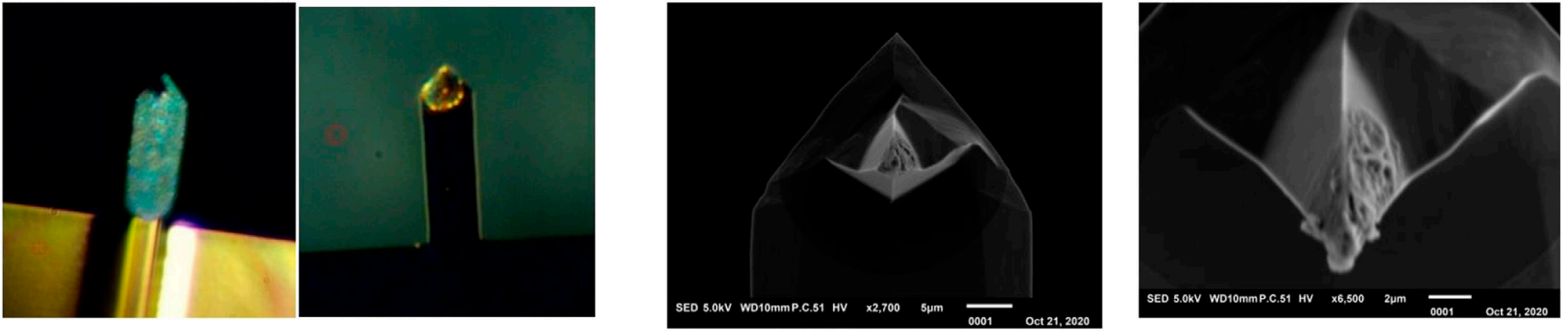
Left: Lithiation covering the cantilever (EC/DMC based electrolyte). Right: Lithiation of the tip by alloying (Ionic Liquid electrolyte).

When the probe is brought very slowly to the liquid surface, a sudden bending of the cantilever is an indication that the tip has made contact with the electrolyte. Electrochemical plating of lithium can be started by applying a potential of a few tenths of volts, which will cause an ion current to flow. In the case of EC/DMC based electrolyte, the main problem is the surface tensile driven fluid flow along the cantilever, as indicated by the dotted line in Figure 4 (middle). Because the forces occurring by this capillary process are very small in relation to the total surface tension of the electrolyte, it cannot be detected whether this takes place. Figure 5 (left) shows two photographs where lithium covers all or partially the cantilever due to electrolyte fluid climbing. It appears that for EC/DMC based electrolyte covering only the tip by immersing in an electrolyte film is difficult to control.

By placing a drop of Ionic Liquid in the center of the lithium disk, a thin film can be created by fast rotation of the substrate. The ionic liquid has a high viscosity and is therefore a fairly viscous layer on the surface of the substrate. By setting a small probe pressure, it is possible to land the probe on the surface without breaking through the liquid. A small potential difference was applied between tip and sample to start lithiation of the NSG30 probe by the alloying method. The results are shown in Figure 5 (right).

#### Electrolyte Soaked Graphite Foil

Pores in electrode material act as capillary channels filled with electrolyte which prevents the solvent from evaporation and form an electrolyte buffer. Figure 4 (right) schematically shows how a porous graphite electrode containing lithium and soaked with electrolyte can be used to lithiate the probe tip. Because lithiation is desired to take place at the probe tip-end, a very small amount of electrolyte is required to create battery operation between the tip and the sample, and due to the capillary forces of the porous graphite channels, this amount remains intact for a very long time.

To provide the graphite electrode with lithium, a dismountable lab-cell was used to form a half-cell by a lithium metal disk (2.011 cm^2^) as the counter electrode, stacked with a separator (Celgard 2,400), filled with 1M LiPF_6_ in 1:1 EC/DMC as an electrolyte, followed by a working electrode of commercial battery graded graphite powder casted on copper sheet (NEI Corporartion, Nanomyte BE200E). The graphite electrode was discharged with 0.1 c (Maccor 4,000) until a potential of 0.1 V against Li/Li^+^ was measured. The graphite foil has been carefully removed from the cell after which it has been applied to the AFMAB cell (Figure 2), provided with electrolyte and connected as a working electrode for lithiation of the probe-tip, as can be seen in Figure 4 (right).

As described above, the use of porous material as a substrate provides advantages. Climbing the electrolyte against the cantilever is counteracted by the capillary forces of the micro channels in the porous material, whereby, after setting the working distance to the sample, only the tip is covered with lithium.

**FIGURE 6:**
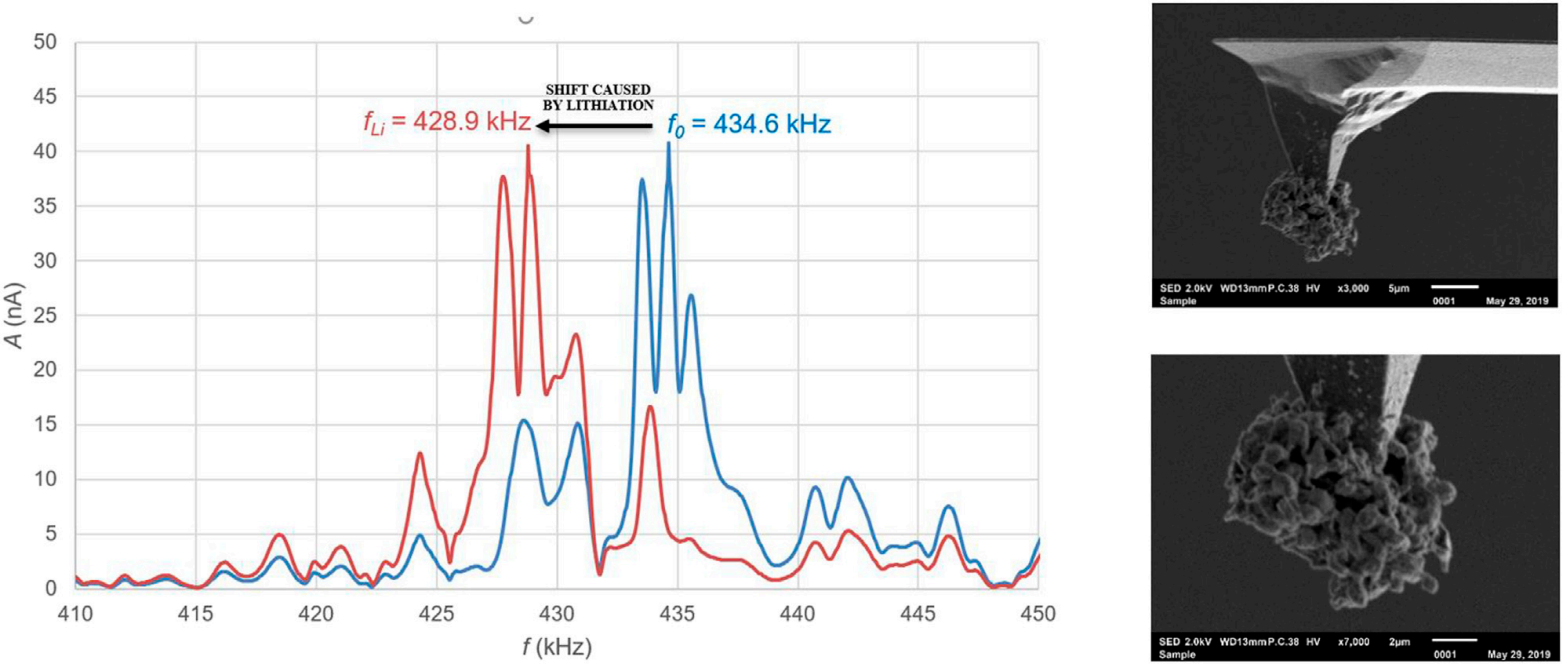
Left: Absolute shift of resonance frequency as a result of lithiation of the tip by electro-chemical plating. Right: A sphere of lithium dendrites at the end of the tip applied by electrochemical plating.

Figure 6 (right) shows a SEM image of a tip that has been treated in this way, where the lithium is deposited only at the very end of the tip. The detailed SEM picture shows that the lithium has formed a sphere of dendrites on the tip. Figure 6 (left) shows the absolute resonance frequency from before and after this treatment, wherein it is clearly visible that the addition of lithium results in a shift of 5.7 ± 0.1 kHz of the resonance frequency. The amount of lithium applied to the tip can be deduced from this shift and is determined on 0.275 ± 0.005 ng.

After applying lithium using the electrochemical plating method, an attempt was also made to electrochemically remove the lithium from the tip back to the substrate. This can be done simply by reversing the potential. This proces has been carried out several times without success which may have to do with the formed lithium dendrites. These dendrites have probably formed a layer of dead lithium on the tip (Chen et al., 2017; Xiao 2019), as a result of which the charge exchange between tip and added lithium is blocked.

### Dry Lithiation

In the dry methods, lithium is transferred to the probe tip without transporting lithium using electrolyte. here two methods have been investigated, a method in which the lithium is applied on a mechanical way and a method in which electro-chemical transport is used.

#### Mechanical Lithiation

**FIGURE 7:**
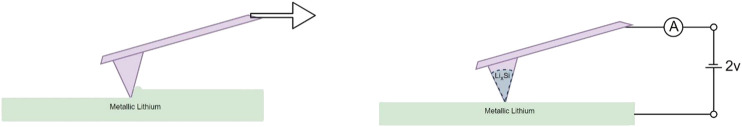
Dry lithiation methods. Left: mechanical lithiation. Right: electrochemical alloying method.

Often a lithographic process occurs when scanning in contact mode on softer materials and surfaces. Scanning the surface with a certain force removes a small amount of material. In some cases, this material will be pushed to the sides of the scanning area, but in other cases, some of the material will stick to the tip. Because lithium is a fairly soft material, it has been investigated here to what extent it is possible to attach an amount of lithium to the tip using this scraping technique. Figure 7 (left) shows a schematic representation of the mechanical lithiation method. The force with which the tip presses on a surface can be set very accurately with AFM equipment. By landing a cantilever probe with a high spring constant on a surface of metallic lithium with a controlled force and then rocking the probe back and forth, the tip of the lithium is scraped off the surface. It turns out that after this operation, some lithium remains on the tip.

For this method, a conductive diamond coated probe with a very high spring constant of typically 80 N/m was chosen (Nanosensors GmbH CDT-NCHR). A metal lithium disc (Sigma Aldrich) was pre-scraped smooth using a sharp doctor blade to remove any oxide layer from the surface, and was then used as the lithium-yielding substrate. To test whether the applied lithium can be electrochemically removed from the tip, a second substrate was positioned next to the lithium disc such that within the x-y plane of the probe control both substrates can be reached. For the second substrate a Silicon wafer (100) was deposited with a layer of gold (∼100 nm) by Magnetron-sputtering (Cressington 208HR, MTM20 thickness controller) and covered with a smooth film of Ionic Liquid.

**FIGURE 8:**
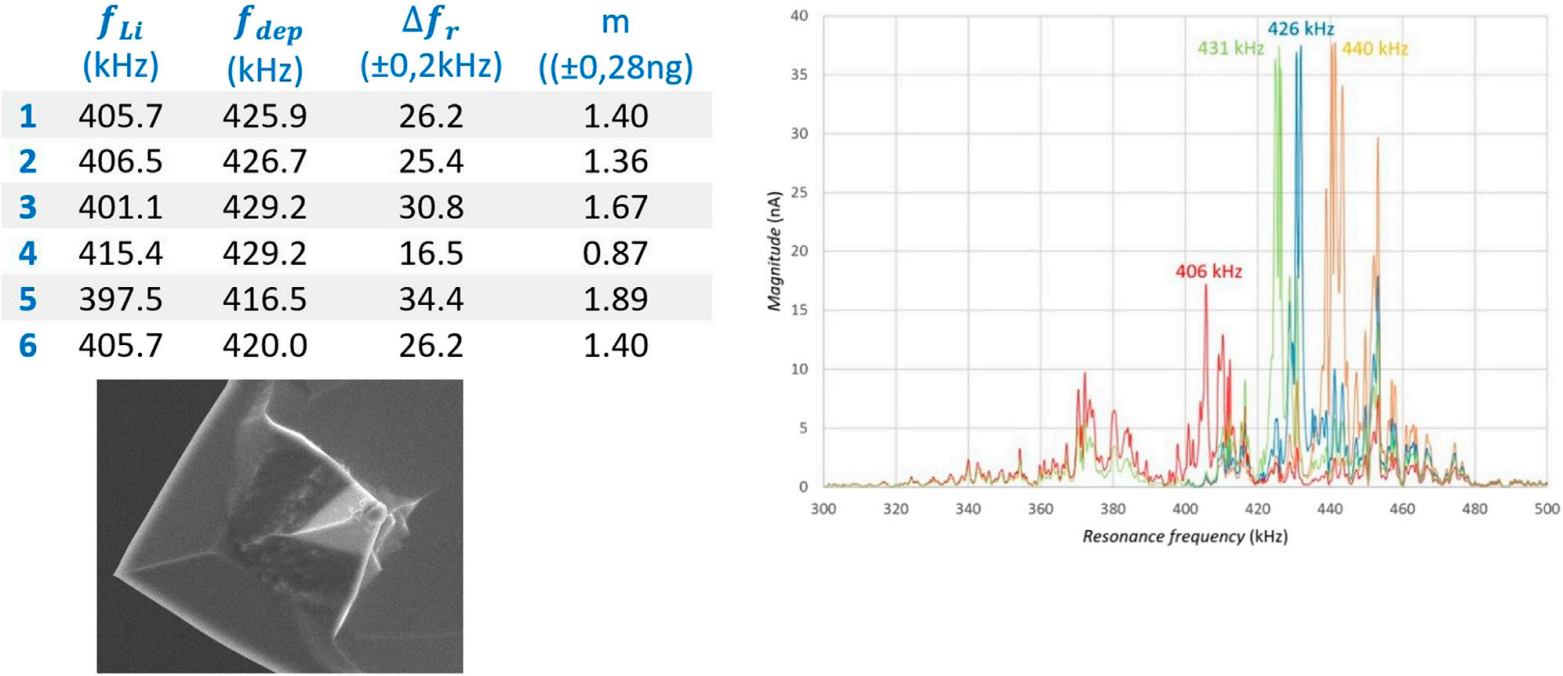
Resonance shift as result of lithiation by using the mechanical method followed by electrochemical de-lithiation: orange = Pristine; Blue, after electrolyte dipping; Red, after mechanical plating on the tip; Green, after deposition back to the substrate.

Referring to Figure 8, which shows the resonance frequency shift for four different situations, the working procedure for applying lithium to the tip and depositing lithium to the substrate is explained. The orange graph shows the frequency spectrum of the pristine and unused probe, with its highest (first order) peak at 440 kHz. First, the probe was brought to the surface of the substrate covered with ionic liquid and the correct height (deposition distance) was sought by gradually lowering the probe until the tip contacts the electrolyte film. When the probe has been lifted until contact with ionic liquid was broken, the free oscillating frequency spectrum was measured. The first order peak has been shifted to a value of 426 kHz (blue line in Figure 10) due to sticking ionic liquid at the tip end. Subsequently the probe was moved to the lithium substrate and was landed with a set force. The probe was moved over the surface back and forth a number of times to collect lithium at the tip by mechanical scraping. After this procedure the free oscillation spectrum was again measured and moved to lower values with the maximum peak shifted to 406 kHz (red line in Figure 10). Finally, the tip was brought back to the substrate covered with ionic liquid and carefully set at deposition distance. A small potential difference between tip and lithium sample was applied to force the lithium deposition to the gold layered substrate. After this, again the free oscillation spectrum was determined, where a shift of the highest peak was encountered to a value of 431 kHz (green line in Figure 10).

To demonstrate the reproducibility of this method, six times an amount of lithium was mechanically applied to the tip and electrochemically returned to the lithium substrate. With the measured resonance shift, the amount of lithium that was electrochemically returned to the substrate was calculated by using the model of 4.4.2. The Table in Figure 8 shows six consecutive measurements, with the measured shift, the corresponding calculated mass of lithium and the calculated standard deviation.

#### Dry Electro Chemical Alloying and Plating

A relatively simple method for lithiating the tip of an AFM probe is achieved by establishing dry contact between a silicon probe tip and a metallic lithium substrate. Contact between these surfaces allows lithium to migrate to silicon, a very slow transfer process which is further limited by the fact that the contact is obtained only by the probe tip area that contacts the lithium substrate. However, the process can be accelerated by applying a potential between the tip and the lithium substrate, as shown in Figure 7 (right). In addition to this, the potential also helps bridge any oxide layer present on the lithium surface.

**FIGURE 9:**
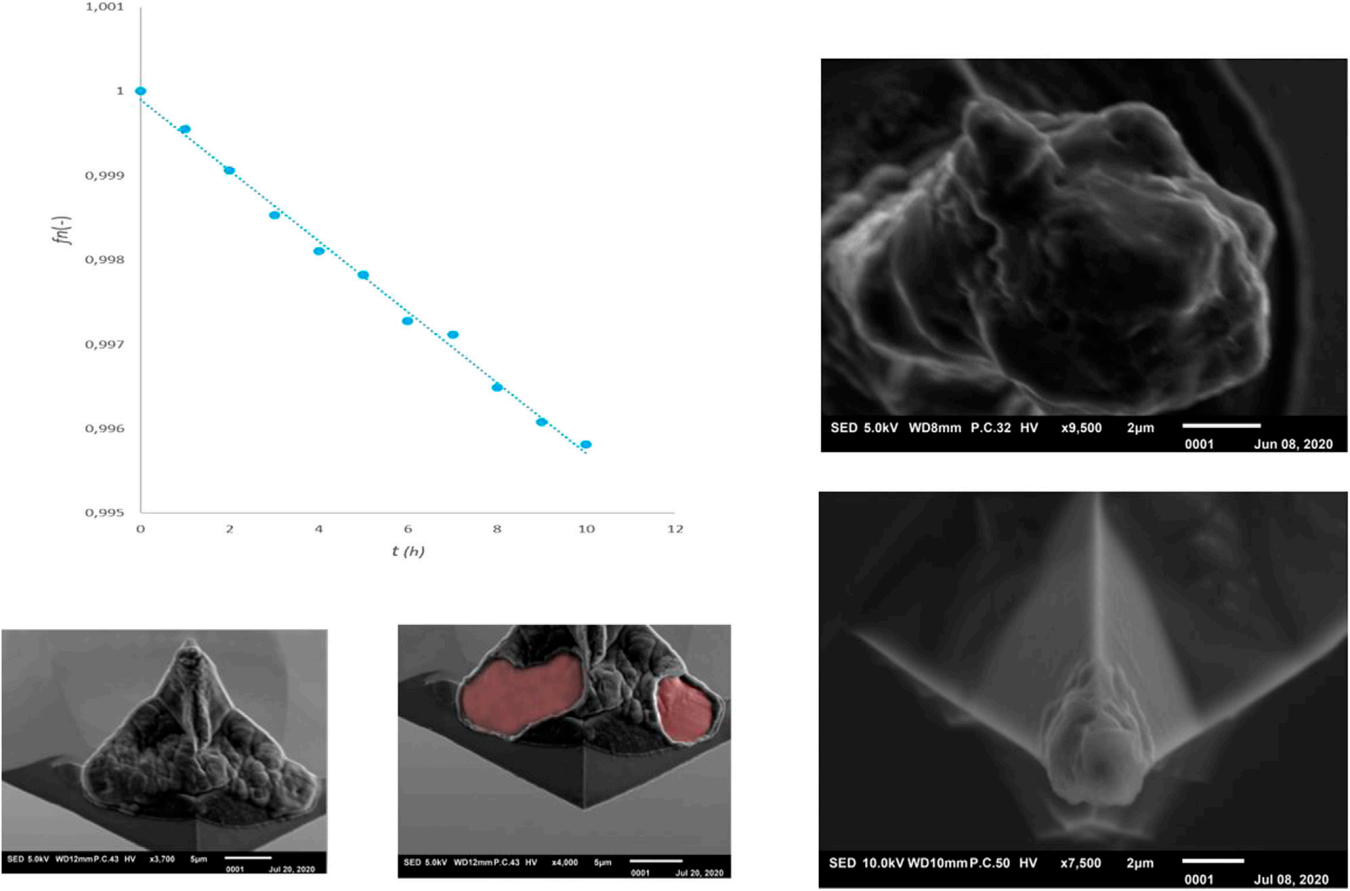
Lithiation of the tip by the dry-alloying **(right)** and the dry-plating method.

A conductive probe of Antimony doped single crystal Silicon (ND-MDT NSG30 N-type 0.01–0.025 mOhm-cm) was selected for dry electro-chemical alloying experiments. The relatively high spring constant (typical 40 N/m) creates a high pressure on the contact surface and the high resonance frequency (measured f_0_ = 269.1505 kHz) enables the use of the frequency shift method for determining lithium mass. In the graph of Figure 9 the shift of the relative resonance frequency f_n_ is shown as a function of the contact time t between the probe and the lithium substrate. During this experiment, the probe was lifted from the substrate every hour to monitor the free resonance frequency and thus the lithiation process. The experiment was conducted for 10 h. Subsequently, the free resonance frequency was followed for three weeks (measuring daily) to determine whether changes were measurable over time. During this period we monitored small variations around the resonance frequency which were comparable with the normal variations of an AFM probe due to long term temperature fluctuations. No permanent shift was observed, so it can be assumed that the probe has remained in the same dynamic state over the three weeks and no major changes have occurred.

Figure 9 (top-right) shows a SEM photo of the probe tip after this lithiation experiment. It can clearly be seen that an alloying reaction has occurred, whereby the tip has expanded enormously. Moreover, the crystal orientation of the tip (100) seems to have created a preferred shape comparable to the structures found in the Nanopillar study of Lee et al. (2011). Despite this successful lithitation, this probe can no longer be used for accurate AFM experiments due to its large dimensions (tip diameter ∼5 micron). It was therefore investigated whether a short alloy-charging can be applied in which the probe tip is minimally deformed. Figure 9 (right-bottom) shows the result of a charge procedure for ∼15 min which results in a shift of the relative resonance to fn = 0.9998. The two extreme situations shown in Figure 9 (right) provide insight into the range within the tip can be lithiated. Depending on the desired scan accuracy, a certain deviation from the tip shape can be accepted and a choice can be made for a degree of lithiation.

The alloying experiments were repeated with a probe of the same composition as used above (Antimony doped single crystal Silicon), but with a lower resonance frequency and spring constant (ND-NDT NSG03, measured f_0_ = 98.971 kHz, typical spring constant 1.74 N/m). These experiments have confirmed that lithium mass determination is less sensitive with a low resonance frequency and the lithiation process is more difficult due to the lower pressure of the tip on the lithium surface.

From various measurements it can be concluded that up to a potential of approximately 2 V between tip and sample (see Figure 9 left-bottom), lithering the tip leads to alloying of silicon with lithium. When the potential is increased, a second process, electro-chemical plating of the probe appears to occur. Figure 9 (bottom left side) shows SEM photos of a tip covered with a layer of lithium. During the SEM analysis, the crust was repelled by charging and parts of the normal tip surface became visible. From this, it can be assumed that the lithium applied by electrochemical plating has poor contact with the underlying probe.

From the performed lithiation experiments it can be concluded that dry lithiation is the most applicable method, with the mechanical method and the alloying method giving the best results (see Table 1).

## Measurements With a Lithiated Probe

### Ion Current Measurements

**FIGURE 10:**
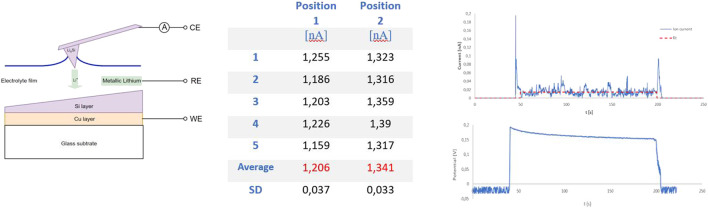
Left: the lithiated probe as an active component in the battery process. Middle and right: Ion current measurements.

The successfully lithiated probes have been used to investigate whether the probe can be used as an active element. Figure 10 (left) schematically shows the measuring principle whereby an electrochemical cell is formed by bringing the probe into contact with a thin layer of electrolyte. In principle, a micro-scale battery has been created between the lithium-containing tip and the sample.

To test the measuring principle of Figure 10 (left), an accurate and flat sample has been obtained by means of sputter deposition techniques. A glass substrate (thickness 175 µ, 30 × 30 mm) is provided with thin layers of copper (current collector) and Silicon (electrode layer). The substrate was cleaned with acetone and isopropanol and placed into a sputter deposition system (AJA Int. ATC 1800) with a pressure of <10^−8^ mBar. After plasma cleaning (5 min in Argon at 20 mBar and RF power of 24 W). First, 15 nm Ti was sputtered to improve adhesion with the substrate, after which a layer of copper of 300 nm was applied (15 min in Argon at 5 mBar and RF power of 100 W). After this, the substrate was set off axes with respect to the target to obtain a thickness gradient in the applied layer. Finally a Silicon layer was deposited (37 min in Argon at 5 mBar and RF power of 100 W) using a mask with a diameter of 2 mm. The thickness gradient of the film was verified using profilometry (DekTak 6 M) and found to be 130–260 nm.

Electrochemical measurements where conducted in the argon filled AFM Glovebox system of 3.1. The sample was placed in the AFMAB cell (Figure 2) and connected as a working electrode. A thin layer of Ionic liquid was applied to the sample surface and spread over the entire area by fast rotating the sampleholder. A small piece of lithium foil was placed on an uncovered glass side of the sample, in contact with the electrolyte to serve as a reference electrode. The electrodes are connected with the tip of the probe acting as the counter electrode, creating a three-electrode configuration. A potentiostat/Galvanostat (Metrohm Autolab PG-STAT302F) was used for measuring the potentials between sample and probe with respect to the lithium reference, while the current has been measured accurately by the i-probe current measurement of the AFM instrument.

We used a lithiated probe prepared by the electrochemical alloy method for ion-current measurements. Two positions with a difference in Silicon thickness have been selected on the sample. We then alternately landed the probe on the layer of Ionic liquid at both measuring points to measure the ion current for approximately 150 s. Because the ionic liquid behaves like a very elastic surface, the gain of the AFM control loop was set at a very low level (0.1) to avoid any unwanted corrections. By lowering the probe step by step, contact was made with electrolyte, after which a potential was immediately measured and an ion current started to flow, see Figure 10. Five separate measurements were taken at each measuring point. The obtained i-probe data was analyzed using *Gwyddion* SPM software (Gwyddion, 2019) for determining the average ion current excluding the start and end spike in the data (see red dotted line in current plot of Figure 10). The two selected measuring points show a significant difference in ion current values, see the table in Figure 10.

### Impedance Measurements Between tip and Sample

The half-cell that can be created between a lithiated tip and an electrolyte covered sample allows very local non-faradaic impedance measurements. The typical probe-to-sample distance is very small due to the use of AFM, so the resolution of this measurement is on the order of the tip-radius. As far as we know, no research is known to determine the impedance as a function of frequency for battery research on this small scale. With the help of local impedance measurements, it is possible to quantify and compare differences in the sample surface and the processes that take place there. In addition, it can contribute to the insights into the origin and composition of the SEI, and it provides opportunities to map impedance data over a surface by combining the measurements with AFM scans. Within the scope of this study, we note that the main goal of our impedance experiments is to demonstrate the possibilities of applying non-faradaic EIS in AFM measurements.

To minimize the influence of the tip on the total impedance, a conductive diamond-coated AFM cantilever with a relatively low resistance was chosen for this experiment (Nanosensors GmbH CDT-NCHR, Nominal force constant 80 N/m, Typical tip resistance 3,000 Ω). The probe was lithiated by the mechanical plating method so in fact a metallic lithium layer covers the tip end. EIS measurements were carried out with the coupled galvanostat/potentiostat (Metrohm Autolab PG-STAT302F). The AFM setup is located within the surrounding glovebox (see *Glovebox Environment*) which functions as a faraday cage and so limits electrical noise. Measurement cables are shielded and cable lengths are balanced and minimized to limit stray capacitance. Preliminary measurements have been made to determine the influence of the cables and the probe on the measurement and it has been found that the contribution of the equipment can be clearly distinguished from signals. Subsequently, a system is measured consisting of an lithium point (the tip), an electrolyte film (1M LiPF_6_ in 1:1 EC/DMC) and a substrate of metallic lithium.

**FIGURE 11:**
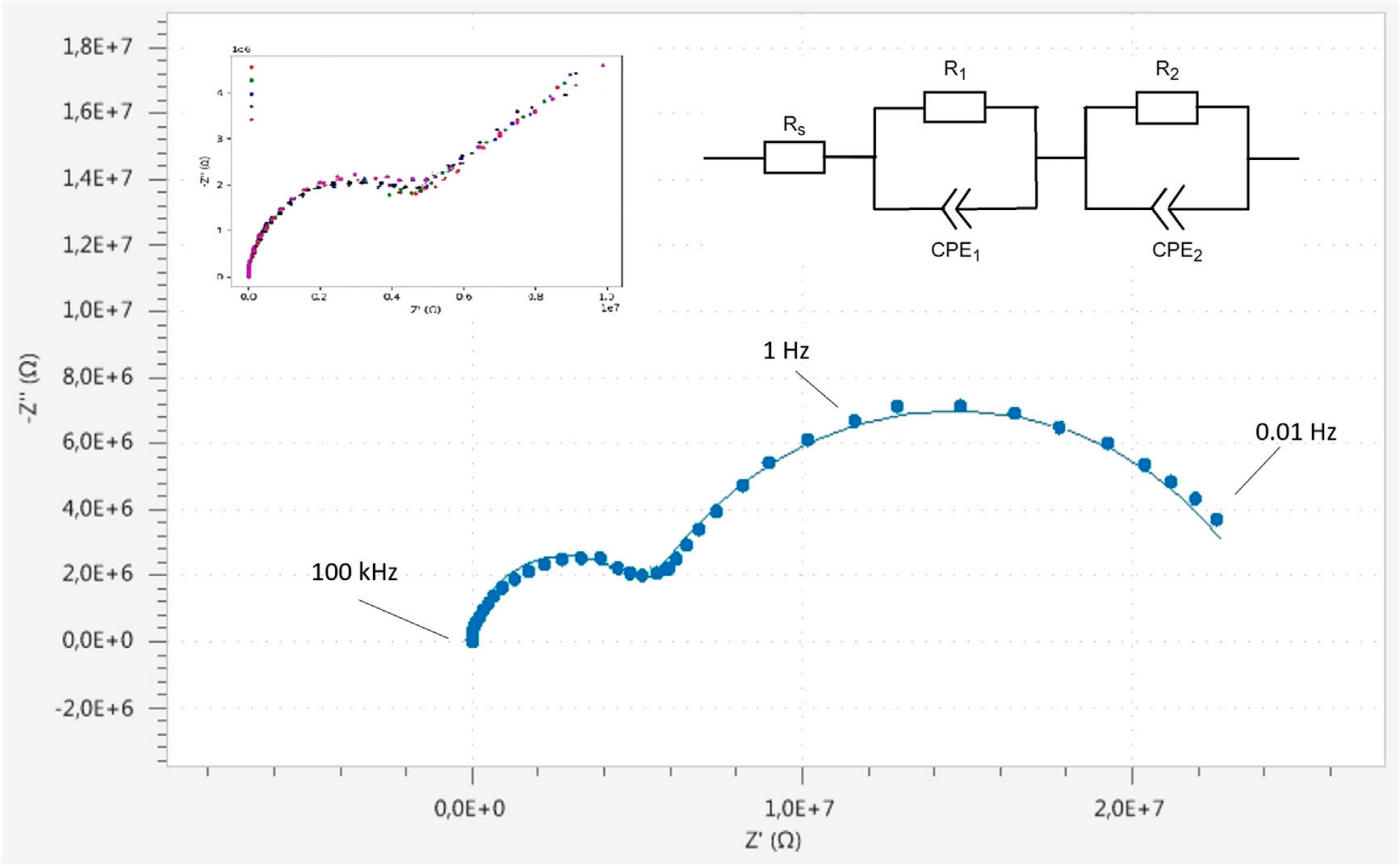
Impedance measurement with ionic contact via liquid electrolyte (1M LiPF6 in 1:1 EC/DMC) between the lithiated tip and a metallic lithium sample. The measurement (00.1 Hz–100 kHz) consist of two semi circles which can be described with the equivalent circuit **(top right)**. Calculated values (NOVA circuit fit, Metrohm Autolab) are Rs = 600 Ω; R1 = 19.5 MΩ; CPE1 = 14.6 nF and *n* = 0.79; R2 = 4.95 MΩ; CPE2 = 252 pF and *n* = 0.96. Five short measurements (1 Hz–100 kHz, **top-left)** show the reproducibility of the measurement technique.

Figure 11 shows Cole-Cole plots of the EIS measurements. To prove the reproducibility, six individual short-time measurements (inset Figure 11 top-left) were carried out within a frequency range of 1 Hz–100 kHz, repeated at the same point and the same sample-probe distance. Subsequently, a longer measurement was taken in which the range was extended to lower frequencies (0.01 Hz–100 kHz), forming two semi-circles in the Cole-Cole plot which may very well be caused by processes at the lithium electrodes (Takami et al., 1992).

The equivalent circuit for this measurement is shown in Figure 11(top-right) and consists of two parallel R/CPE units and an ohmic resistance in series. The electrolyte resistance is represented by R_s_, and charge transfer and typically diffusional processes at both electrodes are given by R_1_/CPE_1_ and R_2_/CPE_2_, respectively. It is however stressed that these processes of both charge transfer and diffusion occur at both electrodes and might be difficult to separate if the characteristic times of the process and geometric factors are close. Since both electrodes are basically lithium and the charge, i.e., the ions, move practically though a similar surface area at both electrodes, the impedances due to both electrodes can be expected to be practically the same, and thus difficult to deconvolute.

## Discussion

The facility described here is suitable for performing *operando* measurements on batteries, in which it is possible to work under very low oxygen, moisture and vibration conditions. A conductive AFM-probe has access to battery electrodes and allows electrochemical measurements in the resolution range of approximately 20–200 nm. To enable measurements in which the probe is an active component of the battery, it has been investigated whether the probe can be supplied with an amount of lithium. Various methods have been investigated whereby the dry lithiation methods give the best results. The lithium applied to a silicon tip by electrochemical alloying can be used in electrochemical measurements and the amount of lithium on the tip can be accurately determined by the occurred shift of the Cantilever natural resonance frequency. By using ionic liquid as electrolyte, the probe can be applied to the tough liquid surface and measurements can be made to determine ion current. In fact, both the charging and the discharging processes (i.e., cycling) can take place between tip and sample. The amount of lithium absorbed into the tip by the alloying method must be carefully monitored as tip distortion occurs at the expense of probe scan resolution.

It appears that lithium can be brought to the tip by wet electrochemical plating, but cannot be removed in a controllable manner. The reverse process whereby lithium is brought from the tip to the sample is blocked by dendrites on the tip end. This layer of dendrite-shaped lithium is formed during the plating process and cannot be electro-chemically returned to the sample (Chen et al., 2017; Xiao 2019).

The mechanical scraping method (moving the needle over a lithium surface with some force) is a good way to provide the tip with lithium. The applied lithium can be removed electro-chemically and can therefore be used as the electrode of a sub-micron battery, whereby only the discharging process can be applied. This method can be used well to make a lithiated tip in a relatively simple way, whereby the amount of lithium can be measured directly with the frequency shift method. The applied lithium is metallic, which offers advantages for some measure possibilities: the so formed *sub-micron half cell battery* between lithiated tip and sample makes non-faradaic impedance measurements possible. Very local impedance can be measured and this information can be mapped and related to a specific sample position.

It is clear that the shift of the resonance frequency of an AFM cantilever is a measure for the mass transport between tip and sample, and can be used to determine the amount of lithium on the tip. The developed FEM model can be a useful tool.

The techniques described here and the results found have demonstrated that it is possible to use this facility and techniques to perform electrochemical experiments on a sub-micron to nano scale. This study shows that the use of lithiated tips offers great possibilities and offers a new addition to operando measurement techniques. The disadvantage is that the measurements are time consuming. However, lithiated tips can be kept in stock without degradation so that series of experiments can be prepared. the study has shown that the principles work. A follow-up study will focus on accurately drawing up assured measurement conditions, making series measurements possible.

The facility offers many possibilities, including research at the SEI, diffusion experiments, local impedance measurements, and measurements on redox processes and ion currents. It is desirable for EIS to be expanded with FFT (comparable to the work of Valiūnienė et al., 2019). This offers possibilities for mapping measurements in which entire impedance spectra can also be recorded. These measurements can be mapped as a function of the location on the sample by combining them with AFM surface scans, and so relationships between morphological and electrochemical properties can be investigated. Measurements are planned for the coming period in which we will use this facility and these methods to determine operando parameters.

### Generalized Conclusion

Despite the technical difficulties, it has proved possible to provide the probe tip of an AFM with lithium and use it as an electrode in a laboratory battery. The resulting “active probe” can be used for non-faradaic ion current and impedance measurements. By demonstrating the measuring principle, the way is cleared to set up an automated 2D mapping method in which topographic, ion current and impedance measurements can be performed simultaneously. The thus created *Electrochemical Active Probe Microscopy* measurement-method can be a new technique for investigating battery processes on a sub-micron to nano scale.

## Data Availability

The datasets generated for this study are available on request to the corresponding author.

## Abbreviations

ac-SECM, alternating current Scanning Electrochemical Microscopy; AFM, Atomic Force Microscopy; AFMAB, Atomic Force Microscope Accessible Battery; DMC, Dimethyl carbonate; EC, Ethylene carbonate; EIS, Electrochemical Impedance Spectroscopy; FEM, Finite Element Method; FFT-EIS, Fast Fourier Transform Electrochemical Impedance Spectroscopy; KPFM, Kelvin Probe Force Microscopy; LiPF_6_, Lithium hexafluorophosphate; SECM, Scanning Electrochemical Microscopy; SCM, Scanning Capacitance Microspopy; SEIM, Scanning Electrochemical Impedance Microscopy; SEM, Scanning Electron Microscopy; UME, Ultra Micro Electrode.
